# Single-Cell RNA Sequencing: Unravelling the Bone One Cell at a Time

**DOI:** 10.1007/s11914-022-00735-w

**Published:** 2022-08-02

**Authors:** Ryan C. Chai

**Affiliations:** 1 Bone Biology Laboratory, Garvan Institute of Medical Research, Darlinghurst, NSW Australia; 2 St. Vincent’s Clinical School, Faculty of Medicine, UNSW Sydney, Kensington, NSW Australia

**Keywords:** Single-cell RNA-seq (scRNAseq), Bone, Microenvironment, Mesenchymal stromal cells (MSCs), Osteoblasts, Osteoclasts

## Abstract

**Purpose of Review:**

Bone is a complex tissue populated by a highly heterogeneous mix of cell types in different compartments. The endosteal compartment is a key site for bone remodelling and provides a supportive microenvironment to harbour haematopoietic and mesenchymal stem cells, as well as cancer cells that grow in bone. The purpose of this review is to summarize recent findings of studies in bone using single-cell RNA sequencing and emergent spatial RNA sequencing to describe different bone-resident cell types and their molecular programs.

**Recent Findings:**

Single-cell RNA sequencing identified novel and transcriptionally distinct cell clusters within different bone cell lineages, including MSCs, osteoblasts, chondrocytes, fibroblasts, osteoclasts and cells of the vasculature. Spatial transcriptomics methods provide information on the localization of the different cell populations.

**Summary:**

Single-cell transcriptomics provided valuable insights into long-standing knowledge gaps in the cellular heterogeneity of bone-resident cells in unprecedented detail, paving the way for studies to further investigate the different cell populations and to develop cell-based therapies for bone diseases.

## Introduction

Musculoskeletal disorders, including osteoporosis and cancers that grow in bone, represent a major burden of disease. The endosteal surface in the skeleton is a main site of bone remodelling, a tightly controlled process involving osteoclasts, which remove bone and osteoblasts, which lay down new bone. These cells are coordinated by osteocytes, which are the master cell regulators in the skeleton. Other cell types and cell states, such as cells of the vasculature and haematopoietic cells, are also likely to contribute to the control of the skeleton. Bone is also a main site for cancer metastasis, with up to 90% of patients with advanced prostate cancer, and 60–70% of breast cancer patients have bone involvement with advanced disease [1]. The success of colonizing cancer cells in establishing themselves in the bone is likely to be determined by intrinsic properties of the tumour cells, which are governed by the primary tumour microenvironment, as well as by acquired characteristics determined by the bone microenvironment [2].

Despite the key role of the endosteal compartment in bone remodelling and disease, the specific identity of the cells that comprise the compartment remains unclear. Traditional tools of assessing the functions of cells within complex tissues rely largely on imaging and functional assessment of genetic models based on single-cell markers to identify cell population of interest. However, there are various unappreciated differences in the cellular composition of bone cells in vitro and these issues potentially extend to in vivo studies using single markers to identify populations of interest, as many of the markers capture not one but multiple cell types [3]. The use of single-cell approaches, in particular single-cell RNA sequencing (scRNAseq), offers the potential to overcome some of these shortcomings. scRNAseq makes it possible to profile the transcriptome of individual cells, thus enabling the unbiased characterization of mixed populations of cells to identify new discrete cellular populations contributing to bone physiology and pathology.

Here we review the insights scRNAseq has provided to our understanding of the endosteal bone compartment. We first describe the different methods of scRNAseq briefly and the cell types identified by these techniques in bone. We then report findings of more recent approaches involving spatially annotated transcriptomics, with limited application in bone so far but holds potential to provide even deeper insight into the cellular makeup of the endosteal bone compartment.

## Methods of Single-Cell Isolation, Library Preparation and Data Analysis

A critical step in conducting a successful single-cell sequencing study of bone is generation of a single-cell suspension of skeletal cells while minimizing the impact on cell viability and transcriptional profile. Optimization of cell isolation procedures must aim to minimize the transcriptional impact of the isolation procedure at the same time that cell yield is maximized, as described in a recent protocol for bone scRNAseq [4].

There are currently two main classes of methods to isolate single cells for RNA sequencing, which vary in the number of cells captured (high-throughput or low-throughput) and how the cells are selected for capture (targeted or untargeted) [5]. The first method, known as Smart-seq, relies on index sorting of cells by FACS into individual wells of 96- or 384-well plates and preparing the RNAseq libraries while the cells are physically separated [6]. The index sorting method allows for the sequencing of full-length transcripts, thus providing improved sensitivity in gene detection. This method is therefore ideal for characterizing individual transcriptome of a small number of cells in detail, rather than interrogating the diversity of a highly heterogeneous pool of cells and identifying rare populations [3]. On the other hand, droplet-based technologies are currently used to capture cells in a high-throughput and unbiased way. The most common platforms include Dropseq, inDrop and the commercially available 10x Chromium system [7–9]. Each of these approaches uses microfluidics to encapsulate individual cells in microdroplets containing single beads with unique barcodes and each mRNA transcript is also marked with a unique molecular identifier (UMI). The cell barcodes enable the assignment of sequence output to individual cells. The UMIs make it possible to eliminate effects of bias associated with PCR amplification from library preparation as reads sharing a UMI are assumed to be derived from the same mRNA molecule. Detailed analysis of the relative strengths of specific methods is available in a recent methodological comparison study [10].

## Bone-Resident Cell Types Identified by scRNAseq

Studies of scRNAseq in bone to date, which contain cells from the endosteal surface and bone marrow, mostly focused on the non-haematopoietic cell types of the murine bone, which provided valuable insights into the heterogeneity of early mesenchymal cells, osteogenic cells, fibroblasts and cells of the vasculature [11–15]. The findings from these studies were mostly coherent, albeit with inconsistent cell nomenclature [16]. More recent studies extended single-cell transcriptomics to profile osteoclast subtypes in vitro and in vivo, identifying novel cell states with potential clinical implications [17, 18]. Here, we aim to describe the cell populations identified collectively to highlight the intracellular heterogeneity and novel subsets of cells that were previously unappreciated.

### Lepr+ and Cxcl12+ Mesenchymal Stromal Cells

Osteogenic cells and adipocytes are believed to derive from mesenchymal stromal cells (MSCs). However, the identification of specific MSC populations remains challenging due to the limited number of specific markers available. scRNAseq studies identified multiple populations of MSC-like cells in bone with high levels of *Lepr* and *Cxcl12* expression, which are characteristics of previously described Cxcl12-abundant reticular (CAR) cells [19]. Lepr+ cells in the bone marrow were thought to overlap with CAR cells, but only with recent scRNAseq technologies was it possible to confirm in detail the cellular relationship between these populations. The Lepr+Cxcl12+ MSCs consisted of mesenchymal cells primed for adipocyte differentiation characterised by high expression of *Cxcl12* and adipogenic markers such as *Adipoq* (adiponectin), as well as cells primed for osteogenic differentiation, with lower expression of *Cxcl12* and high expression of osteogenic genes such as *Sp7* (osterix) and *Alpl* (alkaline phosphatase). The adipo-primed cells were enriched for previously identified genes associated with human bone marrow mesenchymal stem cells [20], suggesting that this might represent a pool of early mesenchymal progenitor cells.

### Osteoblasts, Chondrocytes and Fibroblasts

To infer the hierarchical relationships of MSCs and osteogenic cell types, which include osteoblasts and chondrocytes, directionality of differentiation trajectory was assigned based on changes in transcriptional cell states using pseudotime analysis [21, 22]. Wolock et al. identified a cell cluster with similar profile as the adipo-primed MSCs described in the previous section branching into adipogenic and osteogenic cells along continuous differentiation trajectories [13]. Similarly, Tikhanova et al. and Baccin et al. showed that the differentiation trajectory begins with Lepr+ and Cxcl12+ cells that gradually downregulate adipocytic genes (*Adipoq*, *Lpl*, *Apoe*) and upregulates osteogenic genes (*Sp7*, *Runx2*, *Alpl*) [11, 15].

The first committed osteoblastic cell state identified on the differentiation trajectory is a population of Runx2+ and Sp7+ cells with high expression levels of *Mmp13*, *Postn*, *Alpl* and *Spp1*, all markers of osteoblastic differentiation [12]. However, these cells showed low expression of osteoblast maturation markers, suggesting an intermediate stage before terminal differentiation. Mature osteoblasts identified in scRNAseq have high expression of classic markers such as *Bglap2* (osteocalcin), *Col1a1*, *Sparc* (osteonectin) and *Ifitm5*.

Distinct clusters of chondrocytes were identified by Baryawno et al. due to the high number of non-haematopoietic cells sampled in the study. All identified chondrocytes express chondrocyte lineage genes *Sox9*, *Acan* and *Col2a1* and formed a continuum in the trajectory from pseudotime analysis [12]. The first population identified is the proliferating or resting chondrocytes, expressing highly *Ucma*, a gene detected mostly in the “resting” zone of epiphyseal and vertebral cartilage [23]. Pre-hypertrophic chondrocytes that started the maturation process express *Ihh*, *Pth1r* and *Mef2c* and terminally differentiated hypertrophic chondrocytes express high levels of *Runx2*, *Ihh*, *Mef2c* and *Col10a1.*

Fibroblasts are cells of mesenchymal origin that are ubiquitous in the bone marrow and consist of phenotypically and functionally distinct subpopulations. Multiple fibroblast-like populations characterized by a high expression of extracellular matrix genes were identified, particularly in studies containing digested bones [12, 15]. Fibroblast clusters identified all highly expressed the fibroblast-specific genes *Fn1* (fibronectin-1), *S100a4*, *Dcn* (decorin) and *Sema3c* (semaphorin-3C) and lowly expressed the chondrocyte genes *Sox9* and *Acan*, and were distinguishable from both MSCs and osteoblasts. A cluster expressed progenitor markers *Cd34*, *Ly6a*, *Thy1* and *Cd44*, suggesting these fibroblasts are MSC-like [12]. Additional clusters of fibroblasts, which likely represent cell types deriving from epiphyseal or periosteal locations, express chondrocyte lineage gene Sox9 and the transcription factor *Scx* (scleraxis) that regulate differentiation of tenocytes and ligamentocytes [24]. However, fibroblast-like populations are also present in the bone marrow, as exemplified by the identification of a fibroblast population localizing specifically to arterioles [15, 25].

### Cells of the Vasculature

Bone is a highly vascularised organ with vertically aligned arteries carrying blood from the heart and smaller arterioles feeding into highly branched capillary beds [26]. Capillaries are then drained by sinusoids, which feed into larger veins. These blood vessel networks consist of endothelial cells (ECs) surrounded by smooth muscle cells (SMCs). In line with this, scRNAseq identified arterial and sinusoidal ECs, as well as SMCs as the principal populations of the vasculature in the endosteum and bone marrow. Highly branched sinusoidal capillaries make up the vast majority of vascular cells and arteriole vessels constitute around 10% of the vasculature in bone [11]. Expression of *Flt4*, *Tfpi*, *Stab2* and *Vcam1* are highly specific to sinusoidal ECs and *Cxcl12*, *Kitl*, *Gja4* and *Vegfc* to arterioles. Kusumbe et al. identified CD31^high^EMCN^high^ type-H ECs in the transitioning zones of the metaphysis bridging arterioles with the sinusoids, which have been shown to play a role in osteogenesis [27]. By combining scRNAseq bone datasets, Dolgalev et al. pinpointed a subset of ECs that were not enriched for either arterial or sinusoidal signatures, and express higher levels of *Emcn* and *Cd31* compared to sinusoids, suggesting that they might represent transitional H vessels [16]. This population was found to be enriched for arterial-associated genes *Cd34* and *Ly6c1* but also express unique markers such as *Cotl1* and *Sox4.*

SMCs were found to highly express classical MSC markers Nestin and Ng2 (*Cspg4*) as well as pericyte markers *Acta2*, *Myh11* and *Mcam* [12, 15]. Determining whether Nestin+ and Ng2+ perivascular cells share developmental origins and functional properties with Lepr+ Cxcl12+ MSCs has been a key challenge because there is currently no single marker that separates them [12]. Findings from scRNAseq showed that Nestin+ and Ng2+ SMCs expressed only very low levels of *Lepr*, in contrast to the high expression of *Lepr* in Lepr+ Cxcl12+ MSCs. This suggests that vascular SMCs and MSCs are clearly distinguishable and may represent distinct pools of progenitor cells.

### Osteoclasts

Osteoclasts differentiate from precursors in the monocyte/macrophage lineage. The arrival and attachment of these precursors on bone surface are important steps in the initiation of osteoclast differentiation and bone remodelling. In vitro culture of bone marrow cells with recombinant RANKL and M-CSF can induce osteoclastogenesis, a model that has provided valuable insights into the molecular mechanisms of osteoclast differentiation. However, this culture system contains a highly heterogeneous cell population, which limits our understanding of the precise cell states and genes involved in different stages of osteoclast differentiation. A recent study by Tsukasaki et al. using scRNAseq on osteoclast culture of murine bone marrow cells treated with RANKL and M-CSF was able to overcome this limitation to reveal cell fate decision pathways during osteoclast differentiation in vitro at single-cell resolution [17]. The authors identified multiple clusters of monocytic precursors, which include a cluster of dendritic cell (DC)–like cells expressing *Itgax* (CD11c), a classical DC marker. Deletion of *Rank* (receptor activator of nuclear factor-κB) specifically in CD11c-expressing cells inhibited osteoclast formation in vitro and in vivo. This finding supports previous reports of DCs differentiating into osteoclasts [28, 29]. Two clusters each of pre-osteoclasts and mature osteoclasts were also described, with transcriptional profiles enriched for biological processes involved in membrane raft assembly, proliferation, energy metabolism and terminal differentiation, as well as bone resorption respectively. *Cited2* (Cbp/p300-interacting transactivator with Glu/Asp-rich carboxy-terminal domain 2) was identified to be a key molecular switch triggering terminal differentiation of osteoclasts, and deletion of *Cited2* in osteoclast precursors in vivo resulted in a failure to commit to osteoclast fate. Due to the in vitro nature of this study, contribution of other cell populations to osteoclastogenesis in the native bone marrow microenvironment is still unclear.

In an elegant study using dynamic real-time intra-vital imaging of live mice, McDonald et al. have shown that multinucleated osteoclasts underwent extensive fission to produce daughter cells, a novel cell state called osteomorphs, with the ability to fuse back with the parental network of osteoclasts, a process termed osteoclast recycling [18]. Using scRNAseq, the authors showed that the osteomorphs were transcriptionally distinct from osteoclasts and macrophages. Osteomorphs expressed lower levels of canonical osteoclast markers *Ctsk* and *Atp6v0d2* compared to osteoclasts, and expressed higher levels of surface markers including Axl, Cd11b, Ccr3, Vcam1, Cd74 and Cadm1 when compared to macrophages. The osteomorphs persisted after OPG-Fc withdrawal and exhibited hypersensitivity to RANKL, which drove them to form highly active osteoclasts. Osteoclast recycling therefore not only provides a new paradigm for understanding the behaviour of these cells in their in vivo physiological niche, but also explains the paradoxical acceleration of bone loss and vertebral fractures observed upon discontinuation of the anti-RANKL therapeutic denosumab [30–32] widely used to treat patients with post-menopausal osteoporosis [33] and importantly bone metastases [34, 35].

## scRNAseq Identifies Changes in the Bone Niche During Cancer

Changes within the bone niche during cancer growth and progression, as well as in stress conditions, were also investigated using scRNAseq. Baryawno et al. showed in a murine model of acute myeloid leukemia (AML) that cancer cell growth in bone led to a decrease in mature osteoblasts and a concurrent increase in preosteoblasts, with early MSC numbers remaining stable [12]. This finding suggests a blocking of osteoblast differentiation and a stall at early stages of the lineage during AML expansion. Using a patient-derived xenograft model of AML, Passaro et al. showed that major niche components, including CD31+ cells, Col1a1+ cells and Nes+ cells, which overlapped with signature of endothelial cells, skeletal cells and smooth muscle cells respectively identified in scRNAseq studies, underwent gene expression changes upon AML engraftment [36]. Similarly, Tikhonova et al. reported a decrease in mesenchymal differentiation and a reduced production of pro-haematopoietic factors in the bone marrow of mice treated with chemotherapy [11]. More recently, Zavidj et al. profiled bone marrow immune cells of patients at different stages of multiple myeloma (MM), from precursor stages, monoclonal gammopathy of unknown significance (MGUS), smoldering MM, to full-blown MM [37]. The authors found an early accumulation of regulatory and gamma delta T cells, followed by a loss of CD8+ populations and elevation of interferon (IFN) signaling at later stages. Concurrently, there was a loss of antigen presentation and a T-cell-suppressive phenotype observed in monocytes. Collectively, a better understanding of the changes in the bone microenvironment during cancer using single-cell approaches provides disease hallmarks in the niche, which can potentially serve as novel targets for therapeutic interventions for cancer cells that grow in the bone.

## Spatially Resolved Transcriptomic Approaches

The main limitation of scRNAseq approaches is the loss of spatial context of cells in the tissue being studied. To overcome this issue, spatially resolved transcriptomic imaging approaches have been developed, albeit with limited application currently in bone research. Incorporating laser capture microdissection and bulk RNAseq, Baccin et al. captured and sequenced cells from the arteriolar, sinusoidal and endosteal regions of the bone [15]. Using transcriptomic profiles generated from scRNAseq, the authors found osteoblast and endothelial cell populations were mainly enriched in the endosteum and sinusoid/arterioles respectively. In addition, the adipo-primed MSCs were found to localize preferentially with sinusoidal vasculature, whereas osteo-primed MSCs were found around arterioles and non-vascular regions. Spatial transcriptomics methods are just beginning to reach single-cell resolution, using methods developed recently to measure the spatial distribution of RNA by annealing fixed tissue samples directly to bar-coded primers attached to microscope slides, enabling genome-wide analysis with a resolution of approximately 10 to 30 μm [38, 39]. Using this approach, Tower et al. identified a positive association between sensory axons and mesenchymal progenitor cell fate in a murine model of calvarial suture closure [40]. The inhibition of sensory nerve in vivo reduced proliferative activity of the suture mesenchyme, increased mineral apposition rate along the flanking parietal bone fronts and caused premature suture closure. These findings indicate that sensory nerves are involved in preserving suture patency by delivering soluble factors that support the proliferative capacity of the suture mesenchyme and inhibit downstream differentiation cues, such as BMP signalling. A more recent approach combining photoactivatable fluorescent reporters, two-photon microscopy and scRNAseq was able to infer the cellular and molecular compositions of immune niches, enabling identification of rare niche-specific immune subpopulations and gene programs [41]. The powerful combination of scRNAseq and imaging technologies employed by these methods will greatly improve our understanding of the spatial and temporal aspects of the cellular landscape in bone.

## Outlook and Future Directions

Recent scRNAseq studies have unravelled the complex heterogeneity of cell types present on the endosteal bone surface and bone marrow in unprecedented detail. This has provided valuable insights into long-standing knowledge gaps in the field, including the different cell states of osteogenic and osteoclastic cells, as well as the identities of MSCs. scRNAseq studies also provided evidence of the key role bone niche plays in disease states such as cancer, identifying potential targets amenable to therapeutic intervention.

Despite these advances, there are still limitations in scRNAseq studies with key questions that remained unanswered. The unsupervised nature of clustering cells in scRNAseq analysis involves manual cluster annotation, which limits the reproducibility of the results. This is exemplified by conflicting differential hierarchies of many cell types between studies which led to distinct conclusions despite highly comparable data [16]. Global efforts such as the Human Cell Atlas will be able to remedy this issue by creating references by systematically capturing cell types and states across different tissues [42]. As more bone-specific scRNAseq data becomes available, new data points can be included into a stable reference framework that allows for different levels of resolution and will eventually capture transitional cell states more definitively that fall in between clearly annotated cell clusters [43]. Novel cell states and cell types identified in scRNAseq studies should also be validated functionally using methods such as lineage tracing and imaging approaches to exclude the possibility of tissue processing and analytical artefacts of scRNAseq. Furthermore, critical to our understanding of the functions of identified cell states and cell types is the spatial organisation of cells and their gene expression in bone, profiled at a single-cell resolution. The challenges faced by current spatial transcriptomic methods, including the limits to resolution and sensitivity, as well as throughput and accessibility are being rapidly overcome [44]. Lastly, scRNAseq data and the expansion of protein-protein interaction databases will enable the investigation of intercellular interactions between cells identified in bone, a process intimately linked to bone homeostasis and disease.

In conclusion, scRNAseq is a promising tool for the charting of a comprehensive map of the cellular landscape in bone and their molecular processes, paving the way to the development of novel cell-based therapeutic targets for bone diseases.
